# Low p-SYN1 (Ser-553) Expression Leads to Abnormal Neurotransmitter Release of GABA Induced by Up-Regulated Cdk5 after Microwave Exposure: Insights on Protection and Treatment of Microwave-Induced Cognitive Dysfunction

**DOI:** 10.3390/cimb44010015

**Published:** 2021-12-31

**Authors:** Wei-Jia Zhi, Si-Mo Qiao, Yong Zou, Rui-Yun Peng, Hai-Tao Yan, Li-Zhen Ma, Ji Dong, Li Zhao, Bin-Wei Yao, Xue-Long Zhao, Xin-Xing Feng, Xiang-Jun Hu, Li-Feng Wang

**Affiliations:** 1 Beijing Institute of Radiation Medicine, 27 Taiping Road, Beijing 100850, China; zhi.weijia@163.com (W.-J.Z.); tjuzouyong@163.com (Y.Z.); pengry@bmi.ac.cn (R.-Y.P.); maizhen0906487@sina.com (L.-Z.M.); djtjwj@163.com (J.D.); lillyliz@163.com (L.Z.); ybwcsp@163.com (B.-W.Y.); xlong_z@163.com (X.-L.Z.); 2 Beijing Institute of Pharmacology and Toxicology, 27 Taiping Road, Beijing 100850, China; brenda_echo@sina.com (S.-M.Q.); yht7809@aliyun.com.cn (H.-T.Y.); 3 Endocrine and Cardiovascular Center, Cardiovascular Institute and Fuwai Hospital of Chinese Academy of Medical Sciences, Beijing 100850, China; xinxing_feng@hotmail.com

**Keywords:** microwave exposure, hippocampus, mossy fiber sprouting, GABA, p-SYN1 (ser-553), Cdk5

## Abstract

With the wide application of microwave technology, concerns about its health impact have arisen. The signal transmission mode of the central nervous system and neurons make it particularly sensitive to electromagnetic exposure. It has been reported that abnormal release of amino acid neurotransmitters is mediated by alteration of p-SYN1 after microwave exposure, which results in cognitive dysfunction. As the phosphorylation of SYN1 is regulated by different kinases, in this study we explored the regulatory mechanisms of SYN1 fluctuations following microwave exposure and its subsequent effect on GABA release, aiming to provide clues on the mechanism of cognitive impairment caused by microwave exposure. In vivo studies with Timm and H&E staining were adopted and the results showed abnormality in synapse formation and neuronal structure, explaining the previously-described deficiency in cognitive ability caused by microwave exposure. The observed alterations in SYN1 level, combined with the results of earlier studies, indicate that SYN1 and its phosphorylation status (ser-553 and ser62/67) may play a role in the abnormal release of neurotransmitters. Thus, the role of Cdk5, the upstream kinase regulating the formation of p-SYN1 (ser-553), as well as that of MEK, the regulator of p-SYN1 (ser-62/67), were investigated both in vivo and in vitro. The results showed that Cdk5 was a negative regulator of p-SYN1 (ser-553) and that its up-regulation caused a decrease in GABA release by reducing p-SYN1 (ser-553). While further exploration still needed to elaborate the role of p-SYN1 (ser-62/67) for neurotransmitter release, MEK inhibition had was no impact on p-Erk or p-SYN1 (ser-62/67) after microwave exposure. In conclusion, the decrease of p-SYN1 (ser-553) may result in abnormalities in vesicular anchoring and GABA release, which is caused by increased Cdk5 regulated through Calpain-p25 pathway after 30 mW/cm^2^ microwave exposure. This study provided a potential new strategy for the prevention and treatment of microwave-induced cognitive dysfunction.

## 1. Introduction

With the wide application of microwave technology in medical treatments, military, communications, and many other fields, growing attention has been drawn to its effects on biological systems. The mysterious illness experienced by diplomats from the US embassy in Havana has once again pushed the health hazards of microwave radiation to the forefront [1,2]. Microwaves can be absorbed by organisms, which causes different changes in physiological function such as headaches, neuronal loss, glial cell death, impairments in cognitive functions, abnormalities in neurotransmitters and abnormal blood–brain barrier permeability [3,4,5,6]. Previous studies have indicated that specific doses of microwave exposure can cause decreases in learning and memory [7,8]. Therefore, it is urgent to elucidate the mechanism by which microwaves cause learning and memory dysfunction in order to provide necessary protection and treatment for injuries or dysfunctions caused by microwave radiation.

In the central nervous system (CNS), many intricate electrical processes occur, such as those involved in memory and learning, making them more vulnerable to microwave exposure [6,9,10]. The CNS is known to be one of the most susceptible targets to microwave exposure. The hippocampus is significant in learning and memory, and synapses play an important role in information transfer between neurons.

Cognitive abilities are contingent on a coordinated program of neurotransmitters transferred through presynaptic exocytosis, in which synapsin I (SYN1) plays a key role in regulating the reserve pool of synaptic vesicles in a phosphorylation-dependent manner at the presynaptic terminal [11,12]. In addition, SYN1 can modulate synaptic plasticity by altering the construction of synapses [13,14,15]. SYN1 is phosphorylated at distinct sites by different kinases, among which phospho-sites 4, 5 and 6 (Ser-62, Ser-67 and Ser-549) are phosphorylated by extracellular signal-regulated protein kinase (Erk), while phospho-site 7 (Ser-553) is regulated by cyclin-dependent protein kinases (Cdk5) [16,17,18,19]. The status of the phospho-sites determines the affinity of SYN1 for synaptic vesicles and its capacity to bind to actin, which is important in the regulation of vesicle transportation [20].

The level of p-SYN1 (ser-62/67 and ser-553) was previously observed to fluctuate significantly following microwave exposure, with abnormal release of amino acid transmitters and cognitive dysfunction shown to be induced by microwave exposure [21]. However, the molecular mechanisms associated with these phenomena, as well as whether microwave exposure directly influences the expression of SYN1 and its phosphorylation status or acts through some other upstream molecules such as kinases that promote phosphorylation, have not been elucidated. In this study, the aforementioned question was investigated in order to provide a new strategy for the prevention and treatment of microwave-induced cognitive dysfunction.

## 2. Materials and Methods

### 2.1. Ethics and Methods Statement

All protocols were approved by the Institutional Animal Care and Use Committee of the Beijing Institute of Radiation Medicine. All of the methods in this study were performed in accordance with the relevant guidelines and regulations.

### 2.2. Animals and Groups

190 male Wistar rats (200 ± 20 g) were raised at 20~24 °C with a 50:50 day–night rhythm (lights on at 7 a.m., off at 7 p.m.). Water and food were available ad libitum, and any suffering was minimized as much as possible. The 190 rats were randomly divided into a radiation group and sham radiation group, with 95 rats in each group; 60 rats in each group were randomly selected for the Morris water maze test, and among those rats, after the water maze test 30 rats in each group from each of six time points were selected for Immunofluorescence staining and 15 in each group for Timm staining (three time points). Of the remaining 35 rats in each group, 10 rats in each group were randomly selected for synaptosome extraction in vitro, and the other 25 rats in each group (five time points) were used for protein extraction and subsequent Western blot detection.

### 2.3. Synaptosome Isolation

Unexposed animals were sacrificed, and the hippocampus was quickly removed. Purified synaptosomes were prepared using Percoll gradients as described previously [22], with some minor modifications. The tissue was homogenized in 0.32 M sucrose and buffered at pH 7.4 with Tris. The homogenate was centrifuged (5 min; 1000× *g* at 4 °C) to remove nuclei and debris. The supernatant was gently stratified on a discontinuous Percoll gradient (2, 6, 10, and 20% *v/v* in Tris-buffered sucrose) and then centrifuged at 33,500× *g* for 5 min. The layer between the 10% and 20% Percoll was collected and washed by centrifugation. When used for neurotransmitter release experiments, synaptosomes were resuspended in physiological medium with the following composition (in mM): 125 NaCl, 3 KCl, 1.2 MgSO_4_, 1.2 CaCl_2_, 1 NaH_2_PO_4_, 22 NaHCO_3_, 10 glucose, pH 7.2–7.4.

### 2.4. Cell Culture

PC12 cells were cultured in Dulbecco’s modified Eagle’s medium (DMEM; Invitrogen, Carlsbad, CA, USA) supplemented with 5% 100 U/mL penicillin, 5% (*v/v*) horse serum, 10% (*v/v*) fetal bovine serum, and streptomycin 0.1 mg/mL. Neuronal differentiation was conducted with 20 ng/mL nerve growth factor (NGF) to PC12 cells for 4 d [23].

### 2.5. Microwave Exposure and Dosimetry

As described previously [24], the source of microwaves was a klystron amplifier, model JD 2000 (Vacuum Electronics Research Institute, Beijing, China), which could generate pulsed S-band microwaves at 2.856 GHz in frequency. A rectangular waveguide and an A16-dB standard-gain horn antenna were used for microwave energy transmission in an electromagnetic shield chamber. The average field power density (30 mW/cm^2^) was measured by a waveguide antenna, a GX12M1CHP power meter (Guanghua Microelectronics Instruments, Hefei, China), and GX12M30A power heads.

Rats (or synaptosomes) and cells were randomly assigned into two groups the exposure and sham exposure groups. Animals in the exposure group were exposed to microwaves in a homothermal room, and were placed individually into cages made by polypropylene. Cells were raised at a stationary temperature of 37 °C. The mean power density used for animals and PC12 cell exposure was 30 mW/cm^2^ with the process maintained for 5 min. In order to minimize other psychophysiological effects, experiments for the animals and cells in the sham exposure groups were conducted in the same manner as for the exposed groups, only without microwave exposure.

The finite difference time domain (FDTD) method was applied for SAR (specific absorption rate) calculation. The average whole body SAR value of rats was 14 W/kg in this study, while the SAR value of cells in 6-well plates was 19 W/kg [25]. The hazard threshold for whole body exposure was about 4 W/kg, and increases in core body temperature were of about 1 °C [26,27].

### 2.6. Timm Staining of the Hippocampus

After microwave exposure, a Morris water maze test was performed as reported previously (which showed a decrease in spatial memory performance after microwave exposure) [21]. For Timm staining, rats were anaesthetized with 1% pentobarbital at 7, 14 and 28 d. Subsequently, a heparin PBS solution, 0.4% sodium sulfide solution and 4% paraformaldehyde solution (pH 7.0) was perfused through the left ventricle. Brain tissue was separated and successively placed in 20% and 30% sucrose solutions for gradient dehydration until the tissue sank to the bottom. Coronal sections were obtained using a cryotome. Subsequently, the sections were stained in a mixture of 50% gum arabic, citrate buffer, 5.6% benzene diphenol and 17% argentum nitricum for 20~30 min away from light, after which they were washed in distilled water for 15 min, dehydrated in alcohol xylene, then cleared in xylene and shielded with gelatine. Images of the stained sections were obtained using an optical microscope.

### 2.7. Protein Extraction and Western Blotting

Five rats were anaesthetized by injection of sodium pentobarbital (80 mg/kg) at each time point following microwave exposure. Brain tissues were removed at once, and hippocampi were immediately excised on ice. For the in vitro studies of protein detection, the NGF-induced neurons such as PC12 cells [23] were harvested for protein extraction. Samples of both tissues and cells were homogenated in RIPA buffer with 1% (*v/v*) protease inhibitor cocktail and vibrated for 0.5 h on ice. Then, samples were centrifuged at 12,000 rpm for 15 min (4 °C) and the supernatants stored at −80 °C. Protein quantification was conducted by a bicinchoninic acid protein assay, then the proteins were denatured in 3× sample buffer at 100 °C for 5 min.

### 2.8. Western Blotting

Proteins were fractionated, then the membrane was blocked in 5% low fat milk in PBST at 4 °C overnight and probed with glyceraldehyde-3-phosphate dehydrogenase (GAPDH), p-SYN1 (ser-62/67 or ser-553), Cdk5, Calpain, p35/p25, Erk and p-Erk antibodies. The antibodies were all bought from Santa Cruz Biotechnology; the dilution ratio for GAPDH was 1:10,000, and 1:1000 for the other antibodies. Bands were visualized by FluorChem FC2 (Alpha Innotech, San Leandro, CA, USA) and quantified using Alphaview SA (Alpha Innotech, San Leandro, CA, USA). GAPDH was the internal reference for interested bands in the same sample.

### 2.9. Immunohistochemistry

Tissue slide preparation was performed using the same approaches used for Timm staining. PC12 cells were cultured in six-well plates or in dishes with glass cover slides for 5~7 d. After being successfully induced with NGF (20 ng/mL), the majority of cells were NF positive in the cytoplasm, which extended along the neurite outgrowth. Subsequently, the cells were fixed by methanol and acetone solution (1:1) for 15 min at 4 °C and then fixed to slides with neutral balata and stored at 4 °C.

The slides were rewarmed at room temperature for 30 min and rinsed in PBS for 3 min. After wiping off the water, 0.1% trypsin was added; they were then incubated in a dark box for 5 min. Subsequently, the slides were washed 3 × 3 min times with PBS and incubated with 10% goat serum (diluted with PBS) in a dark box for 15 min. Antibodies against neurofilament protein (NF), p-SYN1 (ser-62/67) and p-SYN1 (ser-553) were all diluted in PBS (1:50) and then incubated at 4 °C overnight. IgG/FITC secondary antibody labelled with green fluorescence protein (1:50 in PBS) was added, and the slides were placed in a dark box in room temperature for 60 min. Finally, the slides were incubated with red fluorescence labelled PI (1:1000 in PBS) and incubated in a dark box at room temperature for 8 min. After mounting the slides with fresh pre-cooled (4 °C) glycerin PBS solution (*v/v* = 1/1), they were observed and imaged via laser confocal scanning microscopy.

### 2.10. Neurotransmitter Release Assays

Synaptosomes were gently shaken and incubated at 37 °C for 0.5 h. The medium of cells or synaptosomes in the sham and exposed groups was changed before microwave exposure in order to normalize the basal level of neurotransmitters, then the synaptosomes and cells were stimulated with 1.5 μg/mL KCl before exposure to microwaves. After 6 h had elapsed following exposure, samples were centrifuged at 12,000 rpm for 5 min, the supernatant was processed by cell-lysis agent and 10% sulfosalicylic acid (*v/v* = 3/2), and then the concentrations of neurotransmitters such as GABA, glycine, aspartate, and glutamate were detected by HPLC.

### 2.11. Amino Acid Neurotransmitter Measurements

Aspartic acid (Asp), glutamic acid (Glu), glycine (Gly), and γ-aminobutyric acid (GABA) were measured by high performance liquid chromatography with a fluorescence detector (HPLC–FLD) procedure. The HPLC system consisted of a microbore reverse-phase column (particle size 5 μm, 150 mm × 4.6 mm; Model Venusil AA, Bonna-Agela Technologies, Shanghai, China), an Agilent 1100 pump (Agilent Technologies, PaloAlto, CA, USA) and a fluorescence detector (Agilent Technologies, USA). The mobile phase (pH 6.8) consisted of 100 mM disodium hydrogen phosphate and 30% methanol; a 1 μL sample was derivated with 5 μL o-phthalaldehyde before being injected into the detection system.

### 2.12. Short Hairpin RNA (shRNA) Transfection of SYN1

The GV175, SYN1 shRNA and control shRNA were constructed using a target sequence (5′ GCAGCTCATCGTGGAACTT 3′) located at the C-terminal coding region, synthesized by Genechem Co. (Shanghai, China). PC12 cells were seeded 8 × 10^5^ cells/well into 60 mm dishes for 24 h before being transfected by plasmid. Transfection was conducted with SYN1 shRNA plasmid in serum-free Opti-MEM with Lipofecamine^TM^ 2000 reagent for 6 h. The transfected cells were maintained in complete medium, then collected at the corresponding time points for use in subsequent analyses.

### 2.13. Inhibition of Mitogen-Activated Protein Kinase Kinase (MAPKK/MEK) and Cdk5 with U0126 and Roscovitine

After PC12 cells were induced by NGF for 5~7 d, MEK and Cdk5 were inhibited using U0126, a common MAPKK inhibitor that can penetrate cells and selectively inhibit MEK1/2, thereby inhibiting the phosphorylation and activation of MAP kinase such as Erk1/2, and Roscovitine, which acts on cyclin dependent kinases Cdc2, Cdk2 and Cdk5 with high efficiency and high selectivity, at a final concentration 10 μM, respectively. Treatment was performed 30 min before microwave exposure. Subsequently, the other experiments were performed as above.

### 2.14. Statistical Analysis

Mean ± standard deviation (SD) was adopted for statistical analyses. Comparisons between the exposure and sham groups were analysed by Student’s *t*-test, while multiple group comparisons were analysed via ANOVA; *p* < 0.05 was considered significant.

## 3. Results

### 3.1. Abnormality in Mossy Fiber Sprouting of the Hippocampus after Microwave Exposure

**Figure 1:**
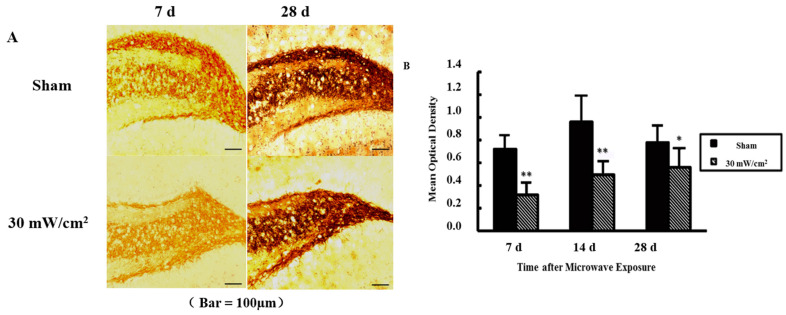
Mossy fiber sprouting of hippocampus after microwave exposure. (**A**) Timm stain of hippocampus after microwave exposure (scale bar = 100 μm); (**B**) Quantitative analysis of Timm stain. (* *p* < 0.05 vs. sham group, ** *p* < 0.01 vs. sham group).

The sprouting of mossy fibers from the dentate gyrus to the hilus and the CA3 region could be observed in the hippocampus of the rats following learning and memory tasks (Figure 1), especially in the sham exposure group, whereas in the exposure group less sprouting was observed. The quantification results showed that at 7 d and 14 d after exposure, the sprouting in the exposure group was weaker compared with the sham exposure group, while at 28 d after exposure, it had recovered to a certain degree.

### 3.2. Expression of SYN1 and Related Proteins in the Rat Hippocampus

**Figure 2:**
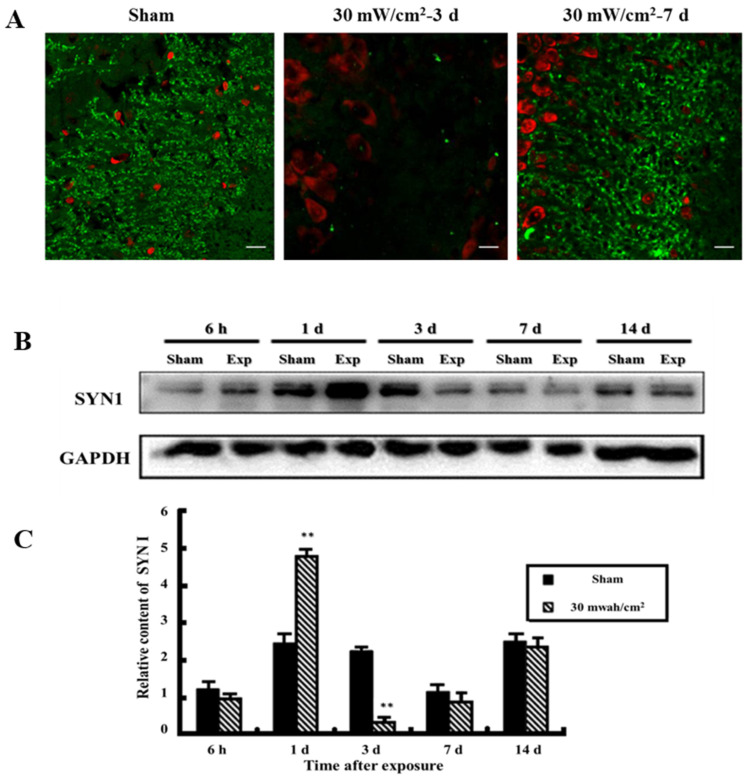
Expression of SYN1 in rat hippocampus. (**A**) In situ expression of SYN1 in hippocampus (scale bar = 50 μm); (**B**) Expression of SYN1 in hippocampus detected by western blot; (**C**) Quantitative analysis of western blot. (** *p* < 0.01 vs. sham group) Gels cropped from different parts of the same gel.

Under normal conditions, there is abundant SYN1 expression in axons and their ends located in the CA3 region and the dentate gyrus. In our study, SYN1 levels decreased compared with the sham exposure group at 3 d and began to increase at 7 d after microwave exposure (Figure 2A).

As shown in Figure 2B,C, expression of SYN1 in hippocampus detected at 6 h~14 d is shown from left to right, successively. At 3 d after microwave exposure, the level of SYN1 was significantly decreased (*p* < 0.01).

**Figure 3:**
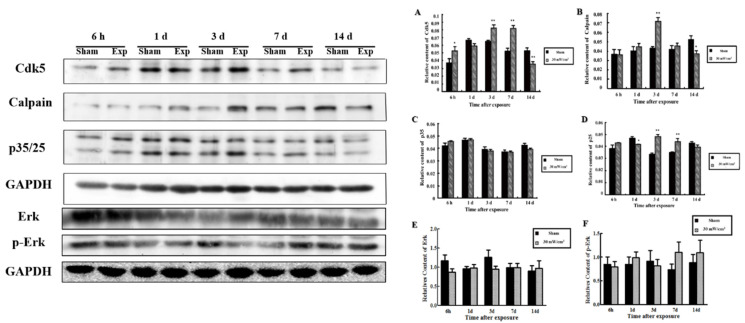
Expression of SYN1 (ser-553) and SYN1 (ser-62/67) related proteins in hippocampus. Expression of SYN1 (ser-553) related proteins such as Cdk5, Calpain, p25 and 35 were detected by western blot; (**A**–**D**) were the quantitative analysis of Cdk5, Calpain, p25 and 35 respectively; Expression of SYN1 (ser-62/67) related proteins such as Erk and p-Erk were detected by western blot; (**E**,**F**) were the quantitative analysis of Erk and p-Erk respectively. (* *p* < 0.05 vs. sham group, ** *p* < 0.01 vs. sham group) Gels cropped from different parts of the same gel.

In Figure 3, the expression of Cdk5, a kinase that regulates the level of p-SYN1 (ser-553), increased significantly at 6 h, 3 d, and 7 d after microwave exposure (*p* < 0.05 or *p* < 0.01); the expression of Calpain increased at 3 d (*p* < 0.01) and then decreased at 14 d after exposure (*p* < 0.05); the expression of p25 exhibited notably lower expression at 1 d (*p* < 0.05), while at 3 and 7 d it was higher than that in sham groups (*p* < 0.01); no significant alteration was observed for p35 expression. The expression of p-Erk, a kinase regulated p-SYN1 (ser-62/67), increased 7 and 14 d after microwave exposure, with the level of Erk also showed corresponding changes.

### 3.3. Alteration of GABA, SYN1 and Its Phosphorylated Proteins in PC12 Cells after Microwave Exposure

**Figure 4:**
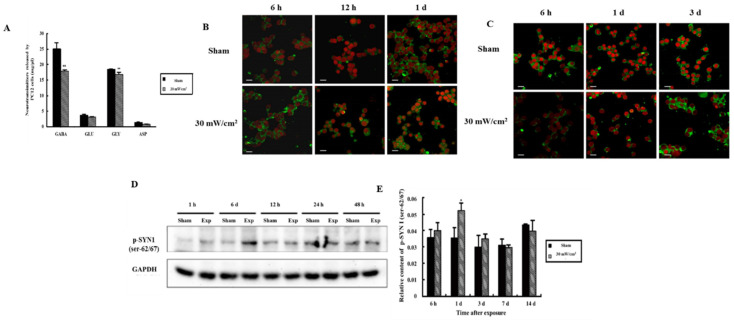
Alteration of GABA, SYN1 and its phosphorylated proteins in PC12 cells after microwave exposure. (**A**) Alteration of neurotransmitters released by PC12 cells after microwave exposure; (**B**) In situ expression of p-SYN1 (ser-62/67) in PC12 cells (scale bar = 50 μm); (**C**) In situ expression of p-SYN1 (ser-553) in PC12 cells (scale bar = 50 μm); (**D**) The expression of p-SYN1 (ser-62/67) was detected by western blot; (**E**) Quantitative analysis of western blot (* *p* < 0.05 vs. sham group, ** *p* < 0.01 vs. sham group) Gels cropped from different parts of the same gel.

In Figure 4A, the level of both GLY and GABA decreased significantly after microwave exposure in PC12 cells, which was consistent with the previously observed changes in the level of these molecules in vivo [21].

For in situ analysis (Figure 4B), p-SYN1 (ser-62/67) was increased at 12 h and recovered at 1 d after microwave exposure, whereas the levels of p-SYN1 (ser-553) (Figure 4C) decreased 6 h after exposure, began to recover at 1 d and then increased significantly 3 d after exposure. Above all, the changes in p-SYN1 in PC12 cells were inconsistent with those observed in the hippocampus [21].

As shown in Figure 4D,E, p-SYN1 (ser-62/67) in PC12 cells increased at 6 h after microwave exposure (*p* < 0.05), while that of p-SYN1 (ser-553) decreased at 6 h and 12 h after microwave exposure and recovered in 2 days [21].

### 3.4. Silencing of P-SYN1 Results in Abnormal Neurotransmitter Release

In order to determine the relationship between the changes in p-SYN1 and decreases in the release of GABA with inhibition of the expression of SYN1, PC12 cells were transfected with SYN1 shRNA. In consideration of microwave exposure and shRNA transfection, PC12 cells were randomly divided into four groups: (1) sham exposed cells transfected with control shRNA (sham + control); (2) microwave exposed cells transfected with control shRNA (expose + control); (3) sham exposed cells transfected with SYN1 shRNA (sham + GV175); and (4) microwave exposed cells transfected with SYN1 shRNA (expose + GV175). Finally, neurotransmitter release by PC12 cells and p-SYN1 expression were detected by HPLC assay and western blot, respectively.

**Figure 5:**
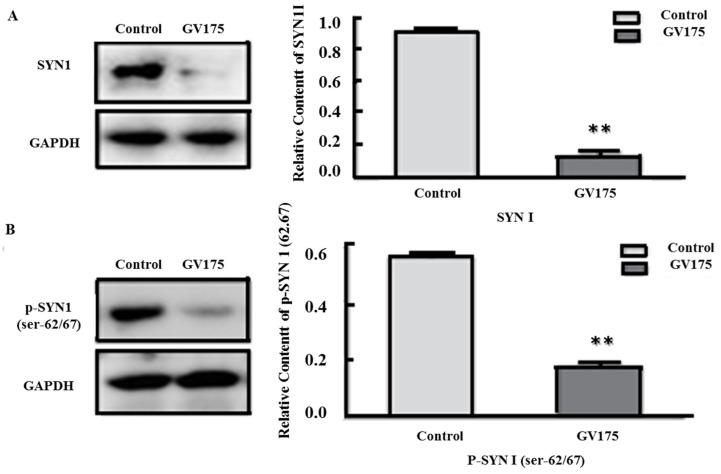
P-SYN1 silencing resulted in the reduction of SYN1 and p-SYN1 (ser-62/67). (**A**,**B**) Expression of SYN1 and p-SYN1 (ser-62/67) in PC12 cells and their Quantitative analysis. (** *p* < 0.01 vs. control group) Gels cropped from different parts of the same gel.

As shown in Figure 5, SYN1 and p-SYN1 (ser-62/67 and ser-553) were observed significantly down-regulated after shRNA transfection (*p* < 0.01) [21]. As reported in our earlier study [21], abnormal release of neurotransmitters such as decreased GABA, GLU and GLY were also induced after transfection.

### 3.5. Alteration of p-SYN1 and Its Related Kinases after Inhibition of MEK and Cdk5

The kinases Cdk5 and MEK are upstream regulators of p-SYN1 (ser-553) and p-SYN1 (ser-62/67), respectively. In MEK inhibition experiments, PC12 cells were assigned to four groups randomly: (1) sham exposure (C + DMSO); (2) microwave exposure (E + DMSO); (3) sham exposure with MEK inhibition (C + U0126); and (4) microwave exposure with MEK inhibition (E + U0126). In Cdk5 inhibition experiments, the groups were similar to those used for MEK.

**Figure 6:**
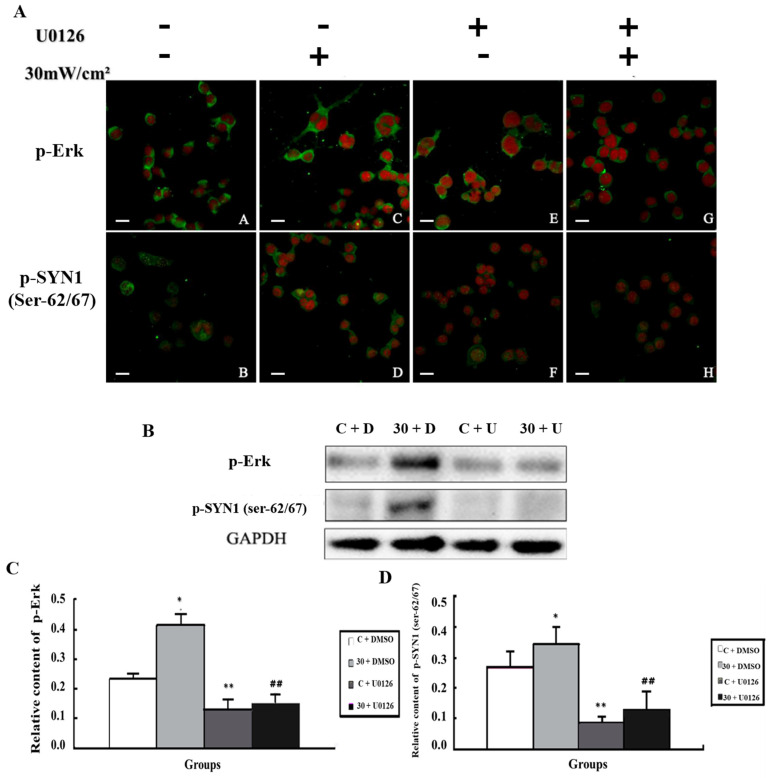
Alteration of p-SYN1 (ser-62/67) and its related kinases after intervention of MEK. (**A**) In situ expression of p-Erk and p-SYN1(ser-62/67) after intervention of MEK; (**B**) Expression of p-Erk and p-SYN1(ser-62/67) detected by western blot; (**C**,**D**) Quantitative analysis of p-Erk and p-SYN1(ser-62/67) in western blot (* *p* < 0.05 vs. control group, ** *p* < 0.01 vs. control group, ^##^ *p* < 0.01 vs. exposure only group) Gels cropped from different parts of the same gel.

In Figure 6, the levels of p-Erk and p-SYN1 (ser-62/67) were up-regulated in the E + DMSO group (*p* < 0.05), whereas when MEK was inhibited by U0126 (C + DMSO), p-Erk and p-SYN1 (ser-62/67) levels were significantly down-regulated (*p* < 0.01). Furthermore, in E + U0126 group, the levels of p-Erk and p-SYN1 (ser-62/67) were also decreased (*p* < 0.01).

**Figure 7:**
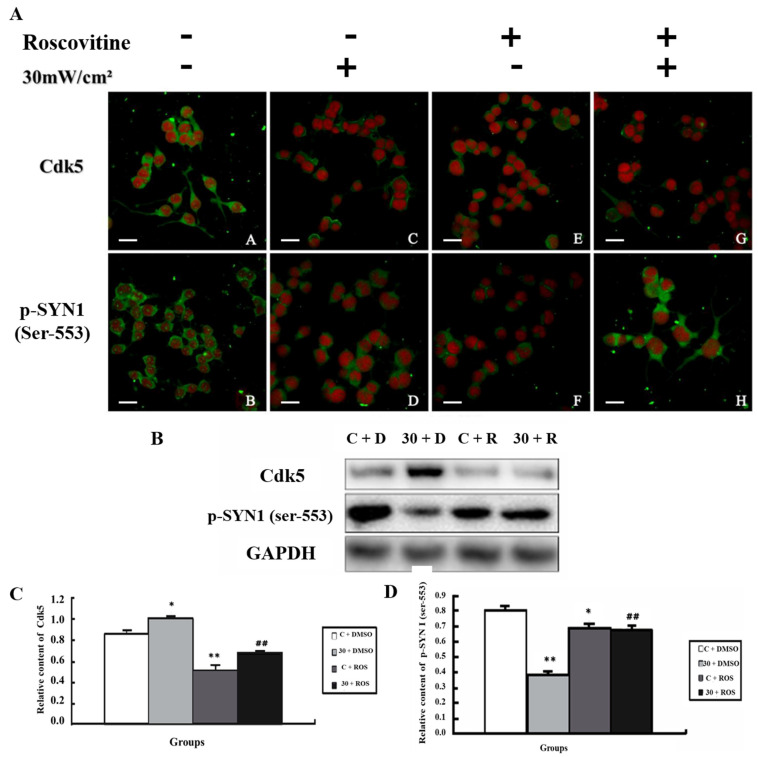
Alteration of p-SYN1 and its related kinases after intervention of Cdk5. (**A**) In situ expression of Cdk5 and p-SYN1 (ser-553) after intervention of Cdk5; (**B**) Expression of Cdk5 and p-SYN1 (ser-553) detected by western blot; (**C**,**D**) Quantitative analysis of Cdk5 and p-SYN1 (ser-553) in western blot (* *p* < 0.05 vs. sham group, ** *p* < 0.01 vs. sham group, ^##^ *p* < 0.01 vs. exposure only group) Gels cropped from different parts of the same gel.

For Cdk5 (Figure 7), its expression increased in the E + DMSO group (*p* < 0.05); when Roscovitine was added (C + Roscovitine), the expression of Cdk5 decreased (*p* < 0.01), and it was lower in the E + Roscovitine group than in the E + DMSO group (*p* < 0.01). P-SYN1 (ser-553) decreased significantly in the E + DMSO group (*p* < 0.01), and showed a higher level in the C + Roscovitine (*p* < 0.05) and E + Roscovitine groups (*p* < 0.01), which was in accord with the expression level in situ.

### 3.6. Alteration of Neurotransmitter Levels after Inhibition of MEK and Cdk5

**Figure 8:**
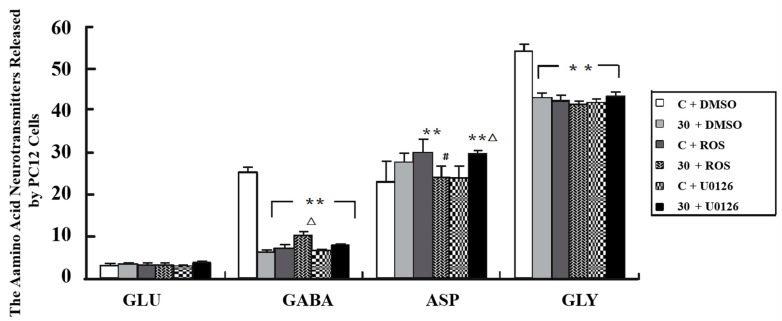
Alteration of Neurotransmitter after Intervention of MEK and Cdk5. The neurotransmitters such as GLU, GABA, ASP and GLY released by PC12 cells after intervention of MEK and Cdk5 were detected and quantized. (** *p* < 0.01 vs. sham group, ^#^ *p* < 0.05 vs. inhibition only group and △ *p* < 0.05 vs. exposure only group).

In Figure 8, the GABA and Gly released by PC12 cells was decreased in the E + DMSO group (*p* < 0.01), and when U0126 or Roscovitine was added, the release of both GLY and GABA was also inhibited (*p* < 0.01). The release of GABA from PC12 cells in the E + Roscovitine group was more than that observed in the E + DMSO group (*p* < 0.05). However, the release of this neurotransmitter by PC12 cells was not influenced by the presence of U0126.

## 4. Discussion

The health hazard of microwave radiation has been a burning issue for many years. Following microwave exposure with the same frequency as in this study, microwave radiation with a power density greater than 10 mW/cm^2^ can lead to a decrease in spatial learning and memory function, inhibition of EEG activity, degeneration of hippocampal neurons, and abnormalities in amino acids and monoamine neurotransmitters; this damage grows more serious as power density increases in a dose-dependent manner [28,29,30].

Learning and memory are advanced functions of the brain which are associated closely with hippocampus. The plasticity of synapses is the neurobiological basis of this process. Long-term potentiation (LTP) of synaptic transmission is a functional indicator of synaptic plasticity in which neurotransmitters act as messengers. Understanding of the abnormal transportation and release of transmitters in presynaptic structures is crucial to elucidate the mechanisms of learning and memory dysfunction caused by microwave exposure; however, few studies have reported on this issue.

Mossy fibre sprouting is a process of synaptic structure remodelling which is closely associated with learning and memory [31,32]. Heimrich et al. studied two different strains of mice and showed that mossy fibre sprouting is related to synaptic transmission and LTP [33]. Regeneration of granule cells and mossy fibres occurred in the dentate gyrus of the hippocampus in adult rats after LTP induction [34]. Mitsuno et al. observed that mossy fibre sprouting occurs in the hippocampal CA3 region of rats after spatial learning and memory through water maze test [35]. Another study showed that under physiological conditions, no mossy fibre sprouted in hippocampus, but sprouting was significantly increased after learning and memory as well as after brain injury. In this study, H&E staining after microwave exposure showed that injury primarily occurred in the dentate gyrus and CA3 region of the hippocampus in rats [21]. Mossy fibre sprouting was observed after learning and memory, while in the microwave-exposed group, the sprouting of mossy fibre was significantly inhibited. Combined with the rats’ deteriorated behaviour in the Morris Water Maze test [21], this suggests that synaptic remodelling is restrained and results in the dysfunction of cognitive ability following microwave exposure.

Most neurotransmitters and neuroactive substances are stored in synaptic vesicles and released through exocytosis. SYN1 regulates the release of neurotransmitters by phosphorylating itself, which regulates the anchoring of synaptic vesicles to the active region in the presynaptic area. SYN1 also plays a role in synaptic plasticity by influencing synaptic structure; in SYN1-knockout mice the presynaptic structure in the CA3 region of the hippocampus and the termini of mossy fibres notably shrank, vesicles docked at the active zone decreased, and the process of sprouting and synapse formation was delayed [12,13]. In a previous report, neurotransmitters were released abnormally in the rat hippocampus, especially attenuated GABA, following microwave exposure. In addition, SYN1 decreased in the hippocampus as did its phosphorylated status, leading to decreased p-SYN1 (ser-553) and increased p-SYN1 (ser-62/67) after microwave exposure, as reported previously; [21] explained the abnormal release of amino neurotransmitters and attenuated mossy fibre sprouting as well as the spatial cognitive ability of rats.

**Figure 9:**
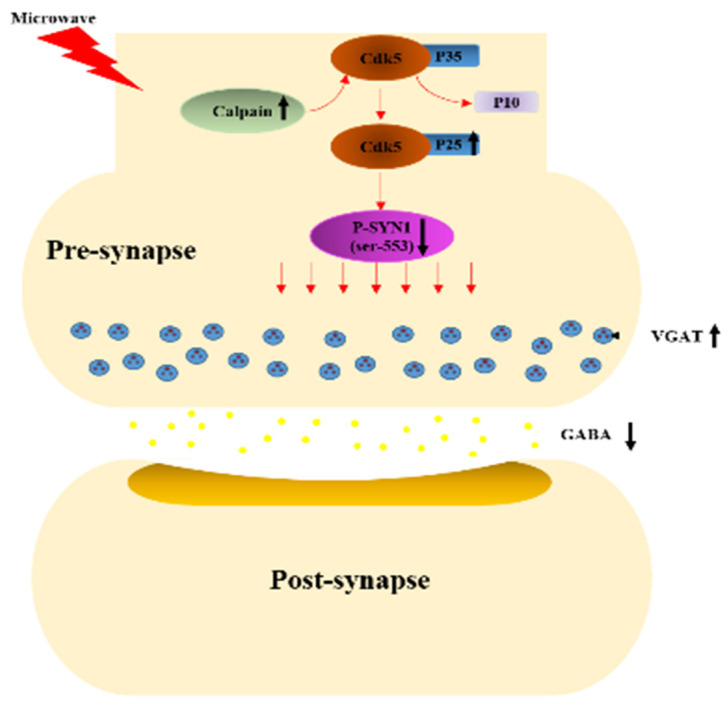
Biological Pathways Impacted by Microwaves. Microwave exposure induces higher levels of Calpain, which subsequently promotes p35 hydrolysis into p25, with increased Cdk5/p25 complex formation, leading to p-SYN1 (ser-553) reduction and abnormal accumulation of VGAT vesicles.

In previous reports, following exposure VGAT and p-SYN1 (ser-553) accumulated in presynaptic vesicles, suggesting that abnormalities in GABA release were regulated by p-SYN1 (ser-553) [21]. Thus, in the present study we investigated the kinase regulating the phosphorylation of this phospho-site, Cdk5, and the upstream molecules Calpain, p25 and p35. Cdk5 was previously shown to be activated during brain development, including migration and development of neuronal axons and dendrites [36]. Recent studies have reported that Cdk5 plays a key role in synaptic plasticity, behavior, and cognition [37,38]. P35, an activator of Cdk5, hydrolyses into p25 and subsequently binds to Cdk5 when Calpain is activated, causing abnormal activation of Cdk5. The Cdk5/p25 complex induces neurotoxicity, cell death and various diseases [39,40,41]. The results of the present study demonstrate the role of the above pathway in the regulation of p-SYN1 (ser-553); that is, microwave exposure induces higher levels of Calpain, which subsequently promotes Cdk5/p25 complex formation, potentially leading to the reduction of p-SYN1 (ser-553) as depicted in in Figure 9. In addition, other mechanisms may participate in the activity regulation of Cdk5; for example, the Erk1/2 pathway, which is mediated by the transcription factors Fos and cAMP-responsive element binding protein (CREB) [42]. In this study, although the total Erk did not change, the detection of Erk1/2 was still required.

To further explore the role of p-SYN1 (ser-62/67) after microwave exposure, we studied p-Erk, which regulates the expression of p-SYN1 (ser-62/67) and exocytosis [21]. Previous studies showed that 30 mW/cm^2^ of microwave exposure can activate the expression of p-Erk through the Ras–Erk pathway, leading to damage to the mitochondrial structure in neurons [43]. The activated Ras–Erk pathway also can induce the formation of p-Erk, promoting the dysfunctional release of neurotransmitters through the phosphorylation of SYN1. In this study, p-SYN1 (ser-62/67) was up-regulated, suggesting that activated p-Erk may cause the observed increase of p-SYN1 (ser-62/67) after microwave exposure, and participate in damaging the learning and memory process. However, the specific mechanism associated with this activity remains to be elucidated.

The results of the in vivo study suggested that SYN1 and its phosphorylation status may be associated with the abnormal neurotransmitter release observed after microwave exposure, with Cdk5 and p-Erk potentially playing important roles in regulating this process. Thus, the observed in vivo effects were subsequently verified in PC12 cells, in which the effects were consistent with the in vivo results. The same effects were also observed when SYN1 was silenced, and the decrease in GABA release was even more significant under the synergy of silenced SYN1 and microwave exposure [21].

To further validate how the SYN1 phosphorylation influences the release of neurotransmitters, inhibitors of Cdk5 and MEK were exerted. For MEK, the levels of p-SYN1 (ser-62/67) and p-Erk showed consistent change in each treatment group; however, the release of neurotransmitters did not change significantly, suggesting that the alteration of neurotransmitters was not impacted by p-Erk but by other mechanisms, and that further exploration is still needed. In contrast, Cdk5 showed a negative effect on p-SYN1 (ser-553), and the alteration in GABA release was consistent with the change of p-SYN1 (ser-553). The above results all suggest that a decrease in the expression of p-SYN1 (ser-553) may result in abnormally low GABA release caused by obstacles in vesicular anchoring induced by increased Cdk5 after microwave exposure.

## 5. Conclusions

In summary, in this study microwave exposure (30 mW/cm^2^) was shown to result in structural damage as well as behavioral and cognitive dysfunction and neurotransmitter (GABA) transmission abnormalities in rats. We showed that abnormal decreases in the p-SYN1 (ser-553) can lead to obstacle in vesicular anchoring of GABA, which is induced by increased Cdk5 activated via the Calpain-p25 pathway following microwave exposure. Although p-Erk may play a role in cognitive impairment after microwave exposure, further investigation of its specific function in this process is still needed. The progress of this study complements previous understanding of the mechanisms by which learning and memory are impaired by microwave exposure, suggesting neurotransmitters and their upstream regulatory molecules play a critical role and thus providing new potential targets and strategies for protecting personnel from microwave exposure.

## Data Availability

The data presented in this study are available on request from the corresponding author. The data are not publicly available due to it will also be used in subsequent studies.
